# Efficacy of Platelet-Rich Plasma in the Treatment of Diabetic Foot Ulcer

**DOI:** 10.7759/cureus.60934

**Published:** 2024-05-23

**Authors:** Muhammad Saim Azam, Muhammad Hassan Azad, Muhammad Arsalan, Ahmed Malik, Raza Ashraf, Hamza Javed

**Affiliations:** 1 General Surgery, CDA (Capital Development Authority) Hospital, Islamabad, PAK; 2 General Surgery, DHQ (District Headquarter) Hospital, Bahawalnagar, PAK; 3 Renal Transplant, St George's Hospital, London, GBR; 4 General Surgery, Shifa International Hospital, Islamabad, PAK; 5 Radiology, Ayub Teaching Hospital, Abbottabad, PAK

**Keywords:** platelet-derived growth factor, gel fibrin, wound care, chronic wound, hyperglycemia, diabetes mellitus, platelet-rich plasma (prp), autologous platelet-rich plasma, chronic non-healing ulcers, diabetic foot ulcers

## Abstract

Introduction

Diabetic foot complications leading to limb amputations pose a global health concern. Platelet-rich plasma (PRP) gel has emerged as a promising method for ulcer healing, leveraging the growth factors provided by autologous PRP to enhance tissue healing. Therefore, we aimed to assess the frequency of the success of PRP therapy in the treatment of non-healing diabetic foot ulcers.

Methods

This quasi-experimental study, conducted in Lahore, Pakistan, from April 2021 to October 2022, enrolled 80 eligible individuals with non-responsive diabetic foot ulcers using a consecutive sampling technique. Inclusion criteria involved patients of both genders, aged 45-75 years, with unhealed diabetic foot ulcers, and exclusion criteria considered factors such as recurrent ulcers at the same site, smoking, and immunosuppressive or anticoagulant drug therapy. Baseline demographic details, ulcer measurements using a scale, and AutoCAD (Autodesk, Inc., San Francisco, California, United States)-assisted quantification of ulcer base were recorded. Autologous PRP injections were administered following strict aseptic protocols, with dressing changes and assessments performed at specified intervals over four weeks. Treatment success, defined as >90% healing after four weeks, was the primary outcome. Data analysis utilized IBM SPSS Statistics for Windows, Version 26.0 (Released 2019; IBM Corp., Armonk, New York, United States), employing post-stratification chi-square and t-tests where appropriate for significant differences.

Results

The mean age of the patients was 60.40 ± 9.72 years, the mean duration of diabetes was 9.48 ± 2.21 years, and the mean ulcer duration was 11.41 ± 1.63 months. The treatment success rate was 63.7%. Age, gender, and disease duration showed no significant impact on treatment success. However, patients with a normal BMI and shorter ulcer duration exhibited a significantly higher success rate (p <0.001 and p = 0.002, respectively).

Conclusions

This study reaffirms the efficacy of PRP in treating non-healing diabetic foot ulcers, aligning with previous research. Despite a slightly lower success rate compared to literature reports, PRP remains a promising agent for managing diabetic foot ulcers.

## Introduction

Diabetes mellitus, characterized by persistent hyperglycemia and metabolic dysregulation, has reached epidemic proportions globally, imposing a substantial burden on healthcare systems. Recent epidemiological data indicate a relentless rise in the prevalence of diabetes, with projections suggesting a further increase in the coming years [1]. Among the myriad complications associated with diabetes, diabetic foot ulcers (DFUs) stand out as a significant concern, affecting up to 15% of individuals with diabetes [2]. This prevalence is particularly alarming in regions like Egypt, where the prevalence of diabetes among those aged over 20 years has steadily increased and is predicted to reach 13.3% by 2025 [1].

The implications of DFUs extend beyond their physical manifestations, encompassing profound effects on the quality of life of affected individuals. DFUs often lead to functional limitations, prolonged healing times, recurrent infections, and psychological distress, collectively contributing to a substantial healthcare burden. The economic impact of chronic wounds, including DFUs, is significant, exceeding $25 billion annually in the United States [3,4]. Chronic non-healing wounds, affecting about 2-2.5% of the United States population, pose a considerable economic burden on healthcare, with costs expected to rise due to factors such as an ageing population, diabetes, obesity, and infection challenges [4]. DFUs correlate with a high mortality rate of nearly 50% within five years, underscoring their significant impact on both quality of life and health outcomes [5,6].

Current approaches to managing DFUs involve a combination of standard wound care, offloading techniques, and surgical interventions. However, these strategies face challenges such as prolonged healing times, recurrent infections, and the associated economic burden [3]. Amidst the quest for innovative solutions, platelet-rich plasma (PRP) therapy has emerged as a promising avenue for managing DFUs. PRP, a biological product derived from the patient's own blood, boasts a rich concentration of platelets, growth factors, and other bioactive substances [7,8]. As evidenced by studies, PRP has demonstrated efficacy in accelerating wound closure and enhancing overall healing outcomes in various clinical contexts [7-9].

Wound healing, a complex and dynamic process, involves the interplay of various cellular and molecular factors [10]. Disruptions in this process can lead to chronic wounds, necessitating novel interventions. As the prevalence of chronic wounds continues to rise, there is an urgent need for innovative and cost-effective therapeutic strategies. To address the challenges posed by DFUs, continued research, investment, and the development of effective wound care strategies are essential. The socioeconomic impact is substantial, necessitating comprehensive wound care strategies. Traditional approaches, such as growth factors and negative pressure wound therapy, have been employed to promote healing, yet challenges persist, including cost, patient compliance, and accessibility [2,11].

PRP, with its ability to stimulate tissue repair and regeneration through growth factors like platelet-derived growth factor (PDGF), epidermal growth factor (EGF), and vascular endothelial growth factor (VEGF) holds promise in this regard [3,7]. With advancements in regenerative medicine, the exploration of PRP as a cost-effective and autologous therapeutic approach presents an opportunity to address the limitations of existing treatments [9,10]. The aim of this study is to rigorously assess the effectiveness of PRP therapy in healing DFUs, offering insights that may shape future clinical approaches and contribute to the ongoing discourse on diabetic wound management.

## Materials and methods

This quasi-experimental study was conducted at the general surgery department of Services Hospital, Lahore, Pakistan, from April 2021 to October 2022. The was approved by the Institutional Review Board of the Services Institute of Medical Sciences (approval number: IRB/2024/1315/SIMS). Individuals aged between 45 and 75 years, irrespective of gender, presenting with DFUs that had not responded to oral and topical medical management were included. Patients with recurrent ulcers at the same site, a history of smoking, or recent anticoagulant or immunosuppressive therapy were excluded. Eligible patients were enrolled through the outpatient department of general surgery. Written informed consent was obtained from patients after explaining the purpose of the research and associated risks and benefits.

Employing a non-probability consecutive sampling technique, a cohort of 80 eligible patients were enrolled who met the specific eligibility criteria for unhealed DFUs, meticulously defined by the presence of granulating or sloughy tissue with purulent discharge, an unpleasant smell, undermined edges, and exposure of bone or tendon. The sample size of 80 cases was calculated with a 95% confidence level (CI), a 10% margin of error, and the expected percentage of success, i.e., 70.83%, with PRP injection in patients with unhealed DFUs [12].

Demographic details, including name, age, gender, body mass index (BMI), duration of diabetes, and duration of diabetic foot, were recorded. In all cases, the patients had a history of non-compliance with diabetes medications. Their blood sugar levels were uncontrolled, as indicated by their glycated haemoglobin (HbA1c) levels. Baseline ulcer measurements were obtained using a measuring scale, and photographic documentation was conducted for subsequent assessments. The quantification of wound surface area and volume was achieved through the utilization of AutoCAD version 19 (Autodesk, Inc., San Francisco, California, United States) software. Subsequently, patients received autologous PRP injections.

PRP was prepared by obtaining approximately 20 ml of blood from each patient adhering to strict aseptic protocols. After centrifugation, the plasma was separated and activated using calcium chloride to prepare approximately 6 mL of PRP. The activated PRP was then injected subcutaneously inside and around the periphery of the ulcer. This protocol was repeated once again on day three. A non-absorbent dressing was used to cover the ulcer area. The dressing was changed each time on post-treatment day three; the wound was irrigated with normal saline and assessed for the presence of any form of infection. Appropriate antibiotic therapy (Augmentin 1000 mg twice a day) was given to each patient for five days initiated at the time of therapy. During admission for the PRP therapy, as per the hospital protocols, the patient's sugar levels were controlled using insulin therapy. Endocrinology consultations were arranged for all the patients before their discharge from the hospital. Therefore, post treatment with PRP therapy, the blood sugar levels were optimally maintained. Following the third day of the second round of PRP, the dressing was changed once a week, and the patients were followed up for a period of four weeks post treatment. Dressing changes, wound irrigation, and infection assessments were conducted at weekly intervals. The primary outcome measure, indicative of treatment success, was defined as achieving greater than 90% healing within a four-week timeframe. Success was determined by re-evaluating ulcer measurements at the conclusion of the four-week period.

Data analysis was conducted using IBM SPSS Statistics for Windows, Version 26.0 (Released 2019; IBM Corp., Armonk, New York, United States), calculating the mean and standard deviation (SD) for continuous variables such as age, BMI, and duration of diabetes and DFU. Categorical variables, namely gender and treatment success, were expressed in frequencies and percentages. Post stratification for age, gender, BMI, and duration of diabetes and DFU, chi-square and student’s t-tests were deployed to discern significant differences between groups, with a predetermined significance level of P≤0.05. The systematic approach employed in participant selection, data collection, and statistical analysis was to enhance the reliability and validity of our findings.

## Results

Table 1 shows the clinical and demographic features of our study participants. The majority of our participants, as shown in Table 1, were female (n=47, 58.8%), aged 45-75 years. Obesity was prevalent in 31 (38.8%), emphasizing the link between higher BMI and DFUs. PRP therapy showed promise, with 51 (63.7%) experiencing successful ulcer healing. Participants, with a mean age of 60.40 ± 9.72 years, reflected diverse age groups benefiting from PRP. The average diabetes duration was 9.48 years, and ulcers persisted for 11.41 ± 1.63 months on average, highlighting chronicity.

**Table 1:** Demographic and clinical features of the participants

Categorical Parameters		n (%)
Gender	Male	33 (41.3%)
	Female	47 (58.8%)
Age Categories	45-55 years	27 (33.75%)
	56-65 years	25 (31.25%)
	66-75 years	28 (35%)
BMI Categories	Normal	28 (35%)
	Overweight	21 (26.3%)
	Obese	31 (38.8%)
Healing of Ulcers	No	29 (36.3%)
	Yes	51 (63.7%)
Continuous Parameters		Mean ± SD
Age (Years)		60.40 ± 9.72
Duration of Diabetes (Years)		9.48 ± 2.21
Duration of Ulcer (Months)		11.41 ± 1.63

Table 2 compares scaler characteristics, including age of patients, duration of diabetes, and ulcers, between the healed (n=51) and not healed (n=29) groups in the study. Age and duration of disease showed no significant differences (p=0.349 and p=0.757, respectively), while the shorter mean duration of the ulcer demonstrated a significantly quicker healing response to treatment (p=0.002).

**Table 2:** Comparison of continuous characteristics of patients based on success of treatment

Continuous Characteristics	Healed (n=51), mean ± SD	Not Healed (n = 29), mean ± SD	p-value
Age (years)	61.18 ±9.695	59.03 ± 9.789	0.349
Duration of disease (years)	9.55 ± 2.072	9.38 ± 2.484	0.757
Duration of ulcer (months)	10.96 ± 1.399	12.21 ± 1.740	0.002

Table 3 compares the frequency of patients in the categories, including gender, age, BMI, and duration of ulcers and disease. The categories of gender, age, and duration of disease did not show a significant impact on treatment success, as there was no significant difference in treatment success between the categories of these parameters. However, notable differences emerged in BMI categories (p=0.001), where a higher proportion of participants with a normal BMI (54.9%) achieved healing compared to their overweight and obese counterparts (13.7% and 31.4%, respectively). Similarly, ulcers of shorter duration demonstrated a significantly quicker response to the PRP therapy (p<0.001).

**Table 3:** Comparison of categorical characteristics of patients based on success of treatment

Categorical Characteristics		Success of Treatment		p-value
		Healed (N=51), n (%)	Not Healed (N=29), n (%)	
Gender	Male	22 (43.1%)	11 (37.9%)	0.649
	Female	29 (56.9%)	18 (62.1%)	
Age Categories	45-55 years	15 (29.4%)	12 (41.4%)	0.553
	56-65 years	17 (33.3%)	8 (27.6%)	
	66-75 years	19 (37.3%)	9 (31.0%)	
BMI Categories	Normal	28 (54.9%)	0 (0%)	<0.001
	Overweight	7 (13.7%)	14 (48.3%)	
	Obese	16 (31.4%)	15 (51.7%)	
Duration of Disease	6-10 years	34 (66.7%)	19 (65.6%)	0.917
	11-14 years	17 (33.3%)	10 (34.5%)	
Duration of Ulcer	9-11 months	35 (68.6%)	8 (27.6%)	<0.001
	12-14 months	16 (31.4%)	21 (72.4%)	

## Discussion

Over the last two decades, emerging cellular therapies such as PRP therapy have gathered considerable attention for their potential use in the field of regenerative medicine as therapeutic agents in a range of chronic conditions and can have an adjunctive role in a standardized, quality treatment plan. The curative properties of PRP rely on the fact that platelets are a physiological reservoir of a variety of growth factors with healing functions that have an active role in tissue regeneration [3].

PRP provides almost all of the growth factors required for healing. It exhibits two important roles in wound healing. Firstly, gel fibrin forms a barrier to prevent bacterial contamination. Secondly, the growth factors from platelets trigger wound healing. Platelets also promote the secretion of biologically active proteins, including growth factors such as PDGF, transforming growth factor (TGF)-β, TGF-β2, and EGF. The release of these growth factors into the wound may create an environment more conducive to tissue repair and could accelerate postoperative wound healing [7, 10]. The role of PRP as a local dressing is to provide the required growth factors locally at the wound area. This role is suggested to be beneficial because DFUs are deficient in growth factors [3].

A recent meta-analysis of the use of PRP therapy in cutaneous wounds showed that, compared to control wound care, PRP facilitated wound healing and the ulcers improved significantly in small, hard-to-heal acute and chronic wounds [13]. In addition, platelets exert antimicrobial activity against some bacteria on the skin, and clinical data shows that the presence of infection is reduced in PRP-treated wounds. Therefore, PRP therapy has several advantages that can provide a practical and effective treatment approach for small, hard-to-heal ulcers [14].

Our study observed a notable predominance of females (58.8%) among the participants aged 45-75 years, providing insights distinct from certain studies indicating a male prevalence in DFU [15,16]. However, it is essential to recognize that these variations may result from our focus on a specific population rather than reflecting true diabetes or DFU prevalence in a broader context. The diverse age distribution in our sample, especially with a majority above 55 years, aligns with the general prevalence of diabetes and DFU in the population [4,10]. While studies report varying mean ages, our findings are consistent with literature suggesting DFU primarily affects individuals in their fifth decade or beyond [10,17]. Participants with a mean age of 60.40 ± 9.72 years represented diverse age groups benefiting from PRP, emphasizing its broad applicability. The average diabetes duration of 9.48 years and an ulcer persistence of 11.41 ± 1.63 months underscored the chronic nature of conditions addressed with PRP therapy. Significantly, our study revealed a noteworthy prevalence of overweight and obese participants, emphasizing the association between higher BMIs and DFUs. This corresponds with existing literature underscoring the impact of elevated BMI on various health conditions, including DFUs [13].

Encouragingly, PRP therapy showcased promise, achieving successful ulcer healing in 63.7% of cases. This aligns with findings from India and Saudi Arabia, reporting healing rates of 73.91% and complete healing in all cases, respectively [18,19]. Another study demonstrated a positive response in 63 out of 65 ulcers, supporting PRP's efficacy in reducing volume and undermining ulcers [14]. Similarly, a study with 24 patients treated with a single PRP dose showed wound healing with reduced size in 8.2 ± 1.9 weeks [12]. The consistent success rates in various studies affirm PRP as a safe, simple, and cost-effective procedure for enhancing wound healing in diverse non-healing ulcers.

Various other studies have presented divergent outcomes regarding the success rate of PRP in addressing non-healing DFUs. Abdelhafez et al. reported an impressive 96% success rate in achieving complete healing with PRP injections for DFUs [20]. Similarly, Salem and colleagues documented a notable success rate of 97.6% in achieving complete healing with PRP injections for DFUs [21]. In another study, Murtuza and colleagues reported a commendable 93.33% rate of complete healing in patients treated with PRP for DFUs [22]. In comparison, our study revealed a somewhat lower success rate for PRP (63.7%), placing it below the rates reported by Abdelhafez et al. and Salem and Tawfik. However, our findings were comparable to those of Suthar et al., who demonstrated a success rate of 70.83% with PRP injections in non-healing DFU patients [12]. These varying success rates underscore the nuanced nature of PRP therapy outcomes, suggesting that multiple factors may influence its effectiveness in different patient populations.

Our results indicated that gender, age, and duration of disease did not significantly impact treatment success, consistent with findings in other studies. Notably, differences in BMI categories were observed, aligning with studies reporting a higher proportion of normal BMI participants achieving healing [15,17,18]. A quicker healing response with a lower BMI aligns with findings emphasizing the importance of weight management in optimizing PRP therapy response.

Similar to other studies [8,13,14], the current study reinforces PRP therapy's positive impact on DFUs across diverse demographics, chronicities, and factors like obesity. A significantly quicker healing response in participants with a shorter mean ulcer duration highlights the importance of early treatment with PRP therapy in predicting favorable outcomes [8]. Studies suggest that PRP is a valuable and versatile therapeutic option for enhancing DFU healing, consistently effective across various patient characteristics, regardless of age, gender, smoking habits, or blood pressure status [8]. Our findings also support PRP's potential efficacy in DFU healing, especially in an ageing population with prolonged diabetes and obesity. However, further research is crucial to validate and refine targeted interventions in diabetic wound care. 

Our study has a few limitations including a small sample size and the lack of a comparison group. While consistent positive outcomes across studies underscore PRP's potential in wound care, acknowledging limitations such as sample size variations, study design differences, and the need for large-scale investigations is crucial. Economic evaluations emphasize the importance of cost-effective wound care solutions, warranting comprehensive research and standardized methodologies [11].

## Conclusions

Our study confirms the effectiveness of PRP therapy in treating non-healing DFUs, with a good success rate. The therapy is particularly beneficial for patients with a normal BMI and shorter ulcer durations, emphasizing the importance of early intervention. The results of the current study support the integration of PRP into DFU management strategies. Future research should aim to further validate these findings through larger, standardized studies, enhancing PRP's clinical application and efficacy in diabetic wound care.
